# Combining blood biomarkers and the German version of the Dementia Screening Questionnaire for Individuals with Intellectual Disabilities (DSQIID‐G) for diagnosing cognitive decline in Down syndrome

**DOI:** 10.1002/alz.71296

**Published:** 2026-03-20

**Authors:** Olivia Wagemann, Charlotte Götz, Elisabeth Wlasich, Katja Sandkühler, Catharina Prix, Anna Stockbauer, Lena Marth, Alexander Jäck, Steffen Halbgebauer, Hayrettin Tumani, Günter U. Höglinger, Johannes Levin, Georg Nübling

**Affiliations:** ^1^ Department of Neurology University Hospital Ludwig‐Maximilians‐University (LMU) Munich Munich Germany; ^2^ German Center for Neurodegenerative Disease (DZNE) Feodor‐Lynen‐Straße Munich Germany; ^3^ Department of Neurology University Hospital Ulm Ulm Germany; ^4^ German Center for Neurodegenerative Disease (DZNE) Oberer Eselsberg Ulm Germany; ^5^ Munich Cluster for Systems Neurology (SyNergy) Feodor‐Lynen‐Straße Munich Germany

**Keywords:** Alzheimer's disease, blood biomarker, Dementia Screening Questionnaire for Individuals with Intellectual Disabilities, Down syndrome, glial fibrillary acidic protein, neurofilament light chain, screening

## Abstract

**INTRODUCTION:**

Individuals with Down syndrome (DS) are at risk for Alzheimer's disease (AD). However, diagnosis remains challenging due to variability of intellectual ability and symptom presentation. To investigate whether serum AD biomarkers enhance accuracy of the German version of the Dementia Screening Questionnaire for Individuals with Intellectual Disabilities (DSQIID‐G), we combined test scores with neurofilament light chain (NfL) and glial fibrillary acidic protein (GFAP) levels.

**METHODS:**

Seventy‐eight DS individuals (49% female) completed the DSQIID‐G; previous cohort data were added for a pooled sample (*n* = 164, 47% female). Serum NfL and GFAP were assessed using the automated microfluid Ella system.

**RESULTS:**

Combining the DSQIID‐G with NfL or GFAP resulted in improved accuracy in every diagnostic subgroup. The Youden index in the pooled samples yielded a cut‐off score at 6.5.

**DISCUSSION:**

The DSQIID‐G is a robust screening tool and its combination with AD blood biomarkers aids earlier identification of individuals requiring further diagnostics for DS‐associated AD.

## Highlights

- The German version of the Dementia Screening Questionnaire for Individuals with Intellectual Disabilities (DSQIID‐G) is a reliable screening tool for suspected cognitive decline in adults with Down syndrome (DS).
- A cut‐off score of 6.5 optimizes DSQIID‐G performance for a sensitivity > 80%.
- Adding neurofilament light chain or glial fibrillary acidic protein to the DSQIID‐G further improves discriminatory power for DS‐associated Alzheimer's disease.
- The combined screening–biomarker approach holds promise for early detection of cognitive decline in DS.

## 1 BACKGROUND

Adults with Down syndrome (DS) face an exceptionally high risk of developing Alzheimer's disease (AD), primarily due to the triplication of the amyloid precursor protein gene leading to early amyloid accumulation in the brain.^1^ By the age of 50, the majority of individuals with DS exhibit correlates of increased cerebral amyloid consistent with advanced AD pathology, and show first signs of tau pathology, in turn triggering successive cognitive decline and loss of everyday functioning.^2^, ^3^ Yet, diagnosing symptomatic DS‐associated AD (DSAD) remains challenging due to great heterogeneity in baseline cognitive performance, varying levels of intellectual disability, atypical presentation of early symptoms compared to sporadic AD (sAD), and comorbidities influencing the clinical presentation.^4^, ^5^ Moreover, a continued lack of awareness concerning the high prevalence of DSAD is reported by stakeholders,^6^ so that changes in everyday functioning might still be attributed to the DS phenotype or psychiatric disorders, a phenomenon referred to as diagnostic overshadowing.^7^ These factors often result in late diagnosis of cognitive decline and dementia, which in turn can delay initiation of symptomatic therapy as well as care‐related adaptations in their everyday environment.

To improve early detection of dementia in this complex population, screening tools like the Dementia Screening Questionnaire for Individuals with Intellectual Disabilities (DSQIID) were developed with a focus on early caregiver‐observed behavioral changes.^8^ Our prior German validation of the DSQIID (DSQIID‐G) confirmed good reliability and sensitivity, but highlighted limitations in specificity and in detecting early stages, especially when relying on caregiver report alone.^9^ Moreover, discrepancies between studies in optimal cut‐off scores raised concerns about consistency, which might be caused by several factors including cohort selection, diagnostic work‐up, and the specific diagnostic criteria applied.^8^, ^9^, ^10^, ^11^, ^12^

Given these challenges, integrating fluid biomarkers could enhance diagnostic accuracy for detecting suspected cognitive decline within DSAD and further distinguish it from forms of secondary cognitive decline such as psychiatric or metabolic conditions, particularly in cases with subtle or ambiguous clinical presentations. In the current study, we extend our earlier work in a larger, well‐characterized DS cohort. By evaluating the combined use of the DSQIID‐G and serum levels of neurofilament light chain (NfL) and glial fibrillary acidic protein (GFAP) we aimed at determining whether the addition of these biomarkers could improve sensitivity and specificity in screening for DSAD compared to the application of the DSQIID‐G alone. As these proteins are involved in neurodegeneration and astrocytic activity, both have been well established as blood biomarkers in DSAD, correlating with clinical presentation and disease progression.^13^, ^14^, ^15^

## 2 METHODS

### 2.1 Study cohort and clinical work‐up

All participants and respective caregivers included in this analysis were recruited through the outpatient clinic of the department of neurology at Ludwig‐Maximilians‐University (LMU) University Hospital Munich, Germany, providing written informed consent, either themselves or by proxy, prior to study inclusion. The study conduction adhered to the Declaration of Helsinki in its latest revision and was approved by the local institutional review board (#535‐15 and #17‐126). Because our enrolments reflect routine clinical referrals to our specialized outpatient clinic, without targeted recruitment, the risk for selection bias is considered very low. However, our cohorts reflect the demographics of adults with DS in Germany, where participants are of predominantly European descent, specifically White/Caucasian. While ethnic diversity within our sample is limited, we sought to ensure inclusivity by recruiting participants from various age groups as well as intellectual disability levels. Yet, the presented sample as a real‐life cohort is potentially subject to our immediate circumstances of cultural context and health‐care system.

Levels of individual intellectual disability (ID) were assessed according to the Diagnostic and Statistical Manual of Mental Disorders, 5th edition (DSM‐V) criteria into mild, moderate, severe, and profound ID based on the individual's best‐ever level of functioning, which was obtained from detailed interviews with caregivers, neuropsychological assessment, behavioral observation, and the review of previous medical records.^16^ Upon the visit of patient and caregiver, a thorough work‐up of the medical history was obtained, followed by a neurological examination and a blood analysis for differential diagnostics (differential blood count; parameters of liver, kidney, and thyroid function; levels of vitamin D/B1/B12). Additional diagnostics such as lumbar puncture and cerebral imaging via magnetic resonance imaging or positron emission tomography were performed in cases with suspected cognitive decline due to AD if feasible. Further, when possible, participants underwent a neuropsychological assessment leveraging the validated German version of the Cambridge Examination for Mental Disorders of Older People with Down Syndrome and Others with Intellectual Disabilities (CAMDEX‐DS), in which a neuropsychological test battery (Cambridge Cognitive Assessment CAMCOG‐DS) assesses the domains of orientation, language, memory, attention, praxis, abstract thinking, and visual perception.^17^, ^18^

Following a predefined diagnostic algorithm,^19^ changes reported in cognition, behavior, and activities of daily living by patient or informant, results of the clinical work‐up and results of neuropsychological assessments were evaluated independently by two neurologists to reach a clinical diagnosis of either asymptomatic status (aDS); mild cognitive impairment in the context of AD (pDS); dementia in the context of AD (sDS); secondary cognitive decline due to preexisting diseases other than DSAD, such as psychiatric disorders (CDsecond); or cognitive decline of unknown etiology (CDunclass). The latter was chosen if comorbidities other than DSAD (e.g., poorly controlled epilepsy) could have equally contributed to the cognitive decline observed, or (rarely), if available information was insufficient for reaching a definite diagnosis (e.g., due to lack of cooperation). For this, raters were blinded to respective DSQIID‐G scores.

**RESEARCH IN CONTEXT**

1. **Systematic review**: The authors reviewed all available literature using traditional databases (e.g., PubMed, Google Scholar). Very few publications explored the performance of the German version of the Dementia Screening Questionnaire for Individuals with Intellectual Disabilities in adults with Down syndrome (DS).
2. **Interpretation**: Our results demonstrate that combining the screening tool with serum neurofilament light chain or glial fibrillary acidic protein significantly enhances discriminatory accuracy for detecting Alzheimer's disease–related decline in DS.
3. **Future directions**: Future studies should validate these findings in larger, longitudinal DS cohorts and evaluate how biomarker‐assisted screening influences clinical decision making, early intervention, and outcomes across diverse care settings.

### 2.2 DSQIID‐G assessments

The DSQIID‐G has been recently validated in its German version by our group,^9^ and, mirroring the original version, comprises 53 items divided in three separate parts covering (1) best‐ever level of the patient, (2) dementia‐related symptoms, and (3) questions regarding functioning and behavior of the patient.^8^ The respective assessment of (2) and (3) aim to capture specific changes relative to the individual's baseline cognitive and functioning level reported in (1). Because the actual DSQIID‐G score is only calculated from (2) and (3), the score is not substantially influenced by the premorbid ID level.

Paper versions of the DSQIID‐G were sent out to caregivers ahead of the clinic visit and collected at the appointment.

### 2.3 Blood biomarker measurements

Serum samples for assessment of NfL and GFAP levels were obtained from non‐fasting participants and collected into ethylenediaminetetraacetic acid (EDTA) tubes, which were successively centrifuged at 2000 × g for 10 minutes. After aliquotation into polypropylene tubes, all samples were stored at −80°C within 30 to 45 minutes after blood draw.

Measurements were performed at the University of Ulm using the microfluid Ella automated immunoassay system (Simple PlexTM NfL and GFAP assay, Bio‐Techne) according to established protocols. *Z* scores were derived from a euploid standard population for NfL^20^ and GFAP.^21^

### 2.4 Statistical analysis

For all subsequent analyses, we investigated a (1) validation cohort of newly recruited individuals with DS as well as a (2) combined cohort, made up of the validation cohort as well as our previously published sample,^9^ to establish test performance in new participants independently, but also to allow for pooling groups thereby maximizing statistical power and result reliability. We therefore report both separate and pooled results to provide transparency and not mask any cohort‐specific results from the validation sample.

All statistical analyses were conducted in R Studio (2023.06.1+524). Demographics were summarized as mean ± standard deviation for continuous variables and count percentage for categorical variables. Group comparisons were, due to non‐normality of residuals, conducted via Mann–Whitney *U* tests for continuous and Fisher exact tests for categorical variables. For pairwise group comparisons, the Kruskal–Wallis test with Benjamini–Hochberg correction was applied. Receiver operating characteristic (ROC) analyses were performed for receiving the optimal cut‐off scores via Youden index for the DSQIID‐G as well as NfL and GFAP concentrations, while further investigations into predictive performance of the DSQIID‐G alone as well as in combination with NfL and GFAP serum levels were carried out via logistic regression. For this, NfL and GFAP were logged due to non‐normality of their residuals. To obtain biomarker cut‐offs conditional on a dichotomized DSQIID‐G cut‐off of < 7 or ≥ 7 points, predictions based on a multivariable logistic regression for probability of symptomatic clinical status (sDS+pDS) were derived with a targeted sensitivity of ≥ 80% via ROC analysis. Conditional biomarker cutoffs in original units were then computed by solving the fitted logistic regression equation for the respective biomarker values. Uncertainty around those biomarker cut‐offs was calculated using non‐parametric bootstrapping (1000 iterations) for 95% confidence intervals (CIs).

Relationships between CAMCOG performance and DSQIID‐G scores as well as NfL and GFAP levels were obtained via Spearman correlations. In all analyses, a *p* value < 0.05 was determined as the threshold for significance.

## 3 RESULTS

### 3.1 Cohort characteristics

Out of the 164 individuals included in this study, 78 were not included in our initial study of the German version of the DSQIID^9^ and thus made up the validation cohort of the current analysis. Out of these, 32 presented with cognitive decline (47%), 15 of which were diagnosed as sDS, and three with pDS. Another 10 were categorized as secondary cognitive decline, with the remaining four receiving no definite etiological diagnosis (Table 1). For the whole sample, 89 participants were asymptomatic (54%), while 10 and 32 were diagnosed with pDS and sDS, respectively.

**TABLE 1:** Demographic information on validation as well as whole study cohort.

	Validation Cohort						Whole Cohort					
	aDS*N* = 46^*^	pDS*N* = 3^*^	sDS*N* = 15^*^	CDsecond*N* = 10^*^	CDunclass*N* = 4^*^	*p* value^**^	aDS*N* = 89^*^	pDS*N* = 10^*^	sDS*N* = 32^*^	CDsecond*N* = 22^*^	CDunclass*N* = 11^*^	*p* value^**^
Age (years)	32 ± 9	42 ± 10	53 ± 6	30 ± 8	49 ± 5	<0.001	30 ± 8	46 ± 10	53 ± 5	29 ± 6	46 ± 8	<0.001
Sex						0.3						0.6
Male	22 (48%)	0 (0%)	10 (67%)	6 (60%)	2 (50%)		44 (49%)	4 (40%)	20 (63%)	13 (59%)	6 (55%)	
Female	24 (52%)	3 (100%)	5 (33%)	4 (40%)	2 (50%)		45 (51%)	6 (60%)	12 (38%)	9 (41%)	5 (45%)	
Co‐morbidities causative for classification of CDsecond												
Depression				1 (1%)						4 (18%)		
Regression syndrome				5 (50%)						9 (41%)		
Suspected Tourette syndrome				1 (1%)						1 (5%)		
Unspecified psychiatric condition				2 (20%)						3 (14%)		
Hypothyroidism				0						2 (9%)		
Sensory impairment				0						1 (5%)		
Obstructive sleep apnoea				0						1 (5%)		
Vitamin B12 deficiency				1 (1%)						1 (5%)		
Side effects of medication				0						1 (5%)		
Late puberty				0						1 (5%)		
Premorbid ID level						0.039						0.021
Mild	26 (59%)	2 (67%)	2 (13%)	4 (50%)	1 (25%)		51 (61%)	7 (70%)	6 (22%)	7 (37%)	4 (44%)	
Moderate	15 (34%)	1 (33%)	12 (80%)	3 (38%)	3 (75%)		29 (35%)	3 (30%)	19 (70%)	11 (58%)	5 (56%)	
Severe	2 (6.8%)	0 (0%)	1 (6.7%)	1 (13%)	0 (0%)		4 (4.8%)	0 (0%)	2 (7.4%)	1 (5.3%)	0 (0%)	
Missing	2	0	0	2	0		5	0	5	3	2	
DSQIID‐G score	5 ± 7	18 ± 8	24 ± 12	24 ± 7	26 ± 13	<0.001	5 ± 7	10 ± 7	25 ± 13	21 ± 10	21 ± 9	<0.001
CAMCOG score (%)	64 ± 17	62 ± 5	40 ± 13	54 ± 16	47 ± 6	<0.001	65 ± 16	61 ± 12	37 ± 14	48 ± 19	47 ± 24	<0.001
Missing	2	0	3	3	2		5	0	5	5	4	
Serum NfL (pg/mL)	18 ± 17	28 ± 13	58 ± 29	18 ± 14	56 ± 34	<0.001	21 ± 30	29 ± 12	58 ± 25	17 ± 9	38 ± 28	<0.001
Missing	4	0	2	3	1		13	2	6	6	4	
Serum GFAP (pg/mL)	6.6 ± 3.6	13.3 ± 4.2	15.7 ± 5.7	5.8 ± 4.9	19.8 ± 10.1	<0.001	7.4 ± 6.0	11.9 ± 5.2	16.1 ± 5.7	6.4 ± 3.5	13.3 ± 8.6	<0.001
Missing	4	1	3	3	1		13	3	7	6	4	

* Mean ± SD; n (%)

** Kruskal–Wallis‐rank‐sum test; Fisher exact test.

Abbreviations: aDS, asymptomatic Down syndrome; CAMCOG, Cambridge Cognitive Assessment; CDsecond, secondary cognitive decline not due to dementia, CDunclass, secondary cognitive decline of unclear etiology; DSQIID‐G, German version of the Dementia Screening Questionnaire for Individuals with Intellectual Disabilities; GFAP, glial fibrillary acidic protein; NfL, neurofilament light chain; pDS, prodromal Down syndrome; sDS, symptomatic Down syndrome.

Within both cohorts, age was significantly different between the groups (*p* < 0.001), with sDS, pDS, and CDunclass exceeding the age of aDS and CDsecond by ≈ 15 to 20 years. Sex was evenly distributed in every sub‐cohort (*p* > 0.05); however, for pDS of the validation cohort, all three individuals included were female. Premorbid ID levels were more broadly distributed in aDS and sDS compared to the other groups in the validation (*p* = 0.039) and the whole (*p* = 0.021) cohort.

DSQIID‐G scores were decidedly lower in aDS in both cohorts, increasing along the spectrum with pDS and sDS, while CDsecond and CDunclass showed similar mean scores to sDS (*p* < 0.001), similarly to CAMCOG scores (*p* < 0.001). NfL as well as GFAP showed highest levels in sDS, while aDS and CDsecond were found on the lower ends (both *p* < 0.001 on both cohorts).

Pairwise comparisons with Benjamini–Hochberg correction of DSQIID‐G scores between the groups in the validation cohort returned significantly lower scores for aDS versus sDS (*p* < 0.001), CDsecond (*p* < 0.001), and CDunclass (*p* = 0.011), but not pDS (*p* = 0.062). No other group comparison exhibited significant differences.

**FIGURE 1:**
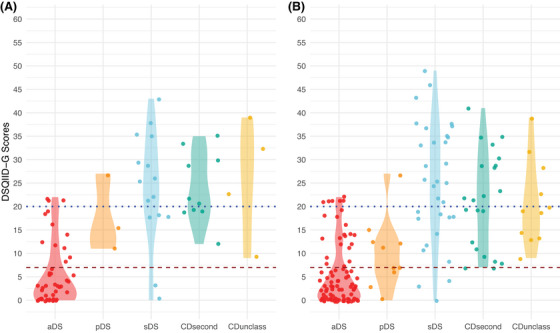
Cross‐sectional DSQIID‐G scores per diagnostic group in the validation (A) and the whole (B) cohort. Horizontal lines correspond to published cut‐off scores at *y* = 20^8^ (dotted line) and *y* = 7^9^ (dashed line). aDS, asymptomatic Down syndrome; CDsecond, secondary cognitive decline not due to dementia; CDunclass, secondary cognitive decline of unclear etiology; DSQIID‐G, German version of Dementia Screening Questionnaire for Individuals with Intellectual Disabilities; pDS, prodromal Down syndrome; sDS, symptomatic Down syndrome.

In the whole cohort, we found significantly lower scores for aDS compared to all other groups (aDS vs. pDS: *p* = 0.034; aDS vs. sDS: *p* < 0.001; aDS vs. CDsecond: *p* < 0.001; aDS vs. CDunclass: *p* < 0.001). Similarly, scores in pDS were significantly lower than those in sDS (*p* = 0.004), CDsecond (*p* = 0.009), and CDunclass (*p* = 0.009), with all other combinations rendering no significant differences in test scores (Figure 1 and Table S1 in supporting information).

### 3.2 ROC analysis

To elaborate on our initial analysis in finding the best cut‐off score for determining cognitive decline due to DSAD when solely using the DSQIID‐G screening tool, we first performed ROC analysis to gather a list of corresponding Youden indices in the present cohorts to obtain the best DSQIID‐G cut‐off for sensitivity > 80% (Tables S2–S5 in supporting information). The sensitivity threshold was chosen due to the concept of the DSQIID as a screening tool, thereby favoring a maximized ability of detecting true positives, and identifying potential false positives in the course of the following clinical work‐up.

For sDS+pDS versus aDS as well as sDS+pDS versus the remaining cohort including aDS, the ideal cut‐off score was 14.5 in the validation cohort (vs. aDS: Youden index = 0.7, sensitivity: 83%, specificity: 87%; vs. rest of cohort: Youden index = 0.53, sensitivity: 83%, specificity: 70%), and 6.5 in the whole cohort (vs. aDS: Youden index = 0.59, sensitivity: 86%, specificity: 73%; vs. rest of cohort: Youden index = 0.39, sensitivity: 86%, specificity: 53%). Additionally, we assessed the ability of the DSQIID‐G to differentiate AD‐related decline specifically from any CDsecond, where we found similar yet overall higher cut‐offs for both cohorts for sensitivity > 80%. However, specificity was, as expected with DSQIID‐G alone, low (Tables S6 and S7 in supporting information).

The same approach was chosen to gather exploratory threshold values for both serum NfL and GFAP, allowing for a specific reference of biomarker concentrations within our cohorts (Tables S8–S15 in supporting information). For the validation cohort, sDS+pDS versus aDS exhibited an NfL cut‐off score of 25.40 pg/ml (Youden index = 0.73, sensitivity: 88%, specificity: 86%), as did sDS+pDS versus rest of cohort (Youden index = 0.66, sensitivity: 88%, specificity: 79%), while the whole cohort exhibited an NfL cut‐off at 24.25 pg/ml for sDS+pDS versus aDS (Youden index = 0.74, sensitivity: 91%, specificity: 83%) and sDS+pDS versus rest (Youden index = 0.71, sensitivity: 91%, specificity: 80%, Tables S8–S11).

With GFAP, sDS+pDS versus aDS exhibited the optimal score at 9.57 pg/ml for the validation cohort (Youden index = 0.81, sensitivity: 93%, specificity: 88%) and at 8.61 pg/ml for the whole sample (Youden index = 0.72, sensitivity: 88%, specificity: 84%). Analyzing sDS+pDS versus rest of cohort we also found a cut‐off at 9.57 pg/ml in the validation sample (Youden index = 0.76, sensitivity: 93%, specificity: 83%), while the whole cohort revealed a value of 8.82 pg/ml (Youden index = 0.69, sensitivity: 88%, specificity: 82%).

### 3.3 Logistic regression for investigation of diagnostic performance

**FIGURE 2:**
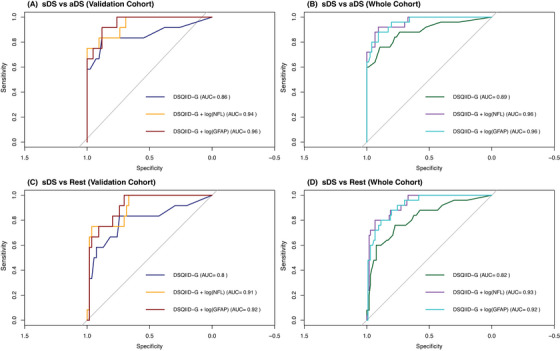
Receiver operating characteristic analysis via logistic regression for the validation (left) and whole (right) cohort investigating the predictive potential of DSQIID, DSQIID+NfL, and DSQIID+GFAP for sDS versus aDS (A&B) and sDS versus the rest of the cohort (C&D), alongside the corresponding AUC estimates. aDS, asymptomatic Down syndrome; AUC, area under the curve; DSQIID, Dementia Screening Questionnaire for Individuals with Intellectual Disabilities; GFAP, glial fibrillary acidic protein; NfL, neurofilament light chain; pDS, prodromal Down syndrome; sDS, symptomatic Down syndrome.

**FIGURE 3:**
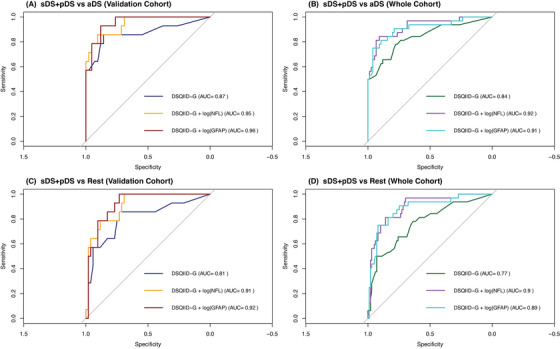
Receiver operating characteristic analysis via logistic regression for the validation (left) and whole (right) cohort investigating the predictive potential of DSQIID, DSQIID+NfL, and DSQIID+GFAP for sDS+pDS versus aDS (A&B) and sDS+pDS versus the rest of the cohort (C&D), alongside the corresponding AUC estimates. aDS, asymptomatic Down syndrome; AUC, area under the curve; DSQIID, Dementia Screening Questionnaire for Individuals with Intellectual Disabilities; GFAP, glial fibrillary acidic protein; NfL, neurofilament light chain; pDS, prodromal Down syndrome; sDS, symptomatic Down syndrome.

Next, we wanted to explore if the addition of NfL or GFAP, both well‐known biomarkers of DSAD‐related pathophysiology,^22^ would increase the diagnostic power of the DSQIID‐G. For this, we investigated sDS versus aDS or versus the whole rest of the cohort, as well as sDS+pDS versus aDS or the rest of the cohort for a more realistic setting within the everyday clinic, in both the validation cohort and the whole sample with blood biomarkers available (Tables S16 and S17 in supporting information and Figures 2 and 3).

In the validation cohort, the DSQIID‐G reached reasonable areas under the curve (AUCs) of 0.86 and 0.8 for differentiating sDS from either aDS or the rest of the cohort, respectively. The incorporation of NfL yielded improvements in AUC of 0.94 (vs. aDS) and 0.91 (vs. rest of cohort) with a particular increase in specificity (96%–100%) but reduced sensitivity of 75%. Adding GFAP instead revealed an even further improvement in AUC (vs. aDS: 0.96; vs. rest of cohort: 0.92) and improved sensitivity (92%–100%), but at the cost of lower specificity (70%–88%). For sDS+pDS versus the rest of the cohort, the DSQIID‐G achieved an AUC of 0.81, which improved with NfL (0.91) and GFAP (0.92), the latter maximizing sensitivity (100%) with modest specificity. Similarly, in sDS+pDS versus aDS, both NfL and GFAP achieved high AUCs (0.95–0.96), with NfL favoring higher specificity (90%).

In the whole cohort, too, all investigated combinations benefited from the incorporation of NfL or GFAP, thereby improving the diagnostic performance compared to DSQIID‐G alone. For sDS versus the rest of the cohort, the DSQIID‐G achieved modest discrimination (AUC = 0.82), while the addition of NfL resulted in the highest accuracy (AUC = 0.93) even more so than GFAP (AUC = 0.92). Similar trends were observed for sDS versus aDS, where NfL again achieved the best performance (AUC = 0.96), closely followed by GFAP (AUC = 0.96). For sDS+pDS versus aDS, with an AUC of 0.84 with DSQIID alone, NfL provided the strongest overall balance (AUC = 0.92, sensitivity: 84%, specificity: 91%), while GFAP maximized specificity (96%) at the expense of sensitivity (75%). Finally, DSQIID‐G performance in sDS+pDS versus rest of cohort amounted to an AUC of 0.77, decidedly increasing with NfL (AUC = 0.9, sensitivity: 81%, specificity: 86%) and GFAP (AUC = 0.89), the latter however markedly favoring specificity (92%) over sensitivity (75%).

To allow for some guidance on biomarker thresholds conditional on the proposed DSQIID‐G score cut‐off at 7 points, we investigated biomarker cut‐off scores using a model‐derived probability threshold corresponding to ≥ 80% sensitivity for sDS+pDS versus rest of cohort. For NfL, a threshold of 47.07 pg/ml (CI: 27.15–125.97) for DSQIID‐G scores < 7 points and a cut‐off at 25.4 pg/ml (CI: 19.84–33.41) for all scores above corresponded with a high risk of clinical DSAD. Analyzing GFAP, we received cut‐off concentrations of 14.30 pg/ml (CI: 9.43–30.31) and 8.11 pg/ml (CI: 6.40–10.36) for DSQIID scores of below and equal or above 7, respectively, associating with a high probability of symptomatic DSAD (Table S18 in supporting information).

Additionally, we repeated the analysis above in sub‐cohorts using previously calculated *z* scores of NfL and GFAP blood levels, thereby referencing a healthy euploid population^20^, ^21^ to account for age‐dependent increases in both biomarkers (Tables S19 and S20 and Figures S1 and S2 in supporting information).

For the validation cohort, we again found moderate discrimination power of DSQIID‐G scores over all combinations (AUC = 0.8–0.87). Incorporating NfL *z* scores in the prediction increased accuracy substantially for all subgroupings (AUC = 0.91–0.95) often at the cost of lower sensitivity but gaining specificity. Similarly, *z* scores of GFAP resulted in AUCs up to 0.96, combining strong sensitivity and specificity, particularly for sDS and sDS+pDS versus aDS.

For the whole cohort, the DSQIID‐G performance was moderate to high, with lowest scores for sDS+pDS (AUC = 0.77–0.89). Again, AUC improved markedly with NfL *z* score incorporation for sDS as well as sDS+pDS (AUC = 0.9–0.99) with balanced sensitivity and specificity. Similarly, use of GFAP *z* scores resulted in an AUC ranging from 0.89 to 0.99 depending on groups investigated, however, favoring specificity.

### 3.4 Spearman correlations

Finally, we assessed the relationship of CAMCOG‐DS performance with DSQIID‐G scores as well as NfL and GFAP blood levels in both cohorts (Figure S3 in supporting information) and found moderate inverse correlations in both cohorts for DSQIID‐G scores (validation: *r* = −0.451, *p* < 0.001; whole: *r* = −0.443, *p* < 0.001) and NfL levels (validation: *r* = −0.422, *p* = 0.001; whole: *r* = −0.425, *p* < 0.001) when related to CAMCOG scores. However, GFAP only exhibited a weak negative relationship in the whole cohort (*r* = −0.3, *p* = 0.001), with the correlation in the validation cohort not reaching significance (*p* = 0.076).

## 4 DISCUSSION

The present study elaborates on our prior work validating the DSQIID‐G by replicating its utility in an independent cohort of newly recruited participants, as well as pooling both cohorts into one large sample. This allowed us to assess DSQIID‐G performance when combined with blood biomarkers and reevaluate the previously determined optimal cut‐off values using the Youden index. Collectively, analyses confirmed the robustness of DSQIID‐G performance across samples, while underscoring the added conceptual and diagnostic value of including blood‐based biomarkers NfL or GFAP. While the DSQIID‐G constitutes a condition‐specific measure of cognitive and/or functional decline in DS, it is not inherently disease specific, resulting in similarly high scores for DSAD compared to CDsecond and CDunclass. Adding serum NfL or GFAP as biomarkers of neurodegeneration or neuroinflammation, respectively, complements this, allowing for a more disease‐specific reflection of ongoing DSAD pathophysiology. This multimodal approach proved able to increase overall accuracy.

The significant correlations of DSQIID‐G, NfL, and GFAP with CAMCOG‐DS performance further support their convergent validity and collective capacity to capture the continuum of cognitive impairment in the context of cognitive decline in DS.

To extend our prior findings on the validation of the DSQIID‐G, we investigated a newly recruited cohort to confirm the potential of the DSQIID‐G as valid screening instrument for suspected cognitive decline in DS.

Across both cohorts, test scores differed significantly between aDS and remaining diagnostic groups, except for pDS in the validation cohort, potentially attributable to its low sample size of three individuals. Yet, these findings reinforce the test's ability to reliably separate asymptomatic DS individuals from those exhibiting cognitive decline.

Building on this framework, we investigated how adding blood biomarkers NfL and GFAP could augment discriminatory power of the DSQIID‐G. While the DSQIID‐G alone demonstrated reasonable discriminatory power, its non–disease‐specific nature limits its capacity to distinguish disease‐related from non–disease‐related cognitive and functional decline. By adding protein biomarkers, the biological specificity provides pathophysiological context to the observed clinical deterioration. In the combined cohort, this resulted in superior specificity and sensitivity, effectively separating sDS from aDS when adding NfL or GFAP, thereby reducing false negatives. For sDS+pDS versus the rest of the cohort, a constellation closer to real‐life populations, the addition of either biomarker showed measurable advantages especially for specificity, potentially facilitating differentiation toward cognitive decline of other causes. Similarly, the use of *z* scores for both biomarkers, as an attempt to control for age‐related increases in biomarker levels, resulted in improved accuracy even exceeding the performance of the unscaled biomarkers in combination with the DSQIID‐G.

These findings support a screening approach in which the DSQIID‐G provides a behavioral and functional index of new cognitive disorder functional impairment, while NfL and GFAP introduce complementary biological context, linking clinical presentation to underlying pathophysiology. Because the DSQIID‐G is a caregiver‐based tool, future applications of such combined measures—for example, by use of dried blood spots—may even allow for the identification of DSAD in settings with limited access to primary and specialist care.

Assessing biomarkers of AD pathology in blood is a minimally invasive and cost‐effective alternative to lumbar puncture and cerebral imaging easily integrated in a routine clinical visit. Considering the increasing number of anti‐amyloid drugs receiving approval in the United States and the EU, there is a need for easy and reliable tools to diagnose DSAD that is not restricted to specialized centers. Plasma NfL has shown promising results for diagnosing DSAD,^13^, ^23^, ^24^ rising ≈ 10 years prior to symptom onset,^25^ while GFAP seems to rise in plasma in the late 20s also showing promising results for DSAD diagnosis,^26^ rendering both proteins a valuable addition to the DSQIID for assessing suspected cognitive decline due to AD in adults with DS. While we focused on providing a refined screening tool rather than a definite diagnostic instrument, the addition of further biomarkers may help reduce the need for cost‐intensive and invasive diagnostic procedures in the future.

In additional ROC analysis, measures of sensitivity and specificity confirmed the previously reported clinically meaningful and reliable range in both cohorts, underscoring the reproducibility of the test's performance across independent samples. Choosing a sensitivity threshold of 80% for the optimal cut‐off in the clinical setting when screening for new cognitive deficits allowed for subsequent diagnostic assessments to identify potential false positives.

Of note, the optimal cut‐off values for a sensitivity of ≥ 80%, derived from performing Youden index analysis, differed between the validation cohort and the combined sample, with a score of 14.5 and 6.5, respectively. Such variability is not uncommon when attempting to define a test‐specific cut‐off score and has been observed across different publications of the DSQIID, too. Specifically, other groups have reported cut‐offs ranging from 5 to 20.^8^, ^9^, ^10^, ^11^, ^12^ For one, this could be due to cohort selection, with some studies recruiting their participants retrospectively based on chart review and previously assigned diagnoses^10^ while others recruited prospectively, thereby being actively involved in the process of reaching a diagnosis.^9^, ^11^ Furthermore, higher cut‐off scores were reported from multiple studies solely leveraging the DSM‐IV^10^ or International Classification of Diseases 10th Revision^8^ diagnostic criteria, both of which have been shown to result in rather low sensitivity in DS patients,^27^ whereas in studies reporting lower cut‐off scores, the diagnostic process involved detailed neuropsychological assessment as well as a case conference at which pDS was specifically considered in individuals not fulfilling clinical dementia criteria.^9^, ^11^

Because diagnostic criteria did not differ between our two cohorts, we mainly attribute this discrepancy to differences in sample size, considering that small increases in sensitivity resulted in considerable reductions of the cut‐off score in the validation cohort (83%: 14.5, 89%: 10, 94%: 2.5). Further, age range and relative distribution of the clinical diagnoses investigated could potentially shift parameter balance in the validation group toward a higher cut‐off. Therefore, the pooled cut‐off values, derived from the largest sample to date in our language, may serve as pragmatic cut‐off score when screening for new cognitive decline in DS. However, this variability remains a practical challenge for clinical application, in which a singular universal cut‐off might not apply across diverse populations and settings. In practice, we suggest applying the proposed threshold of 7 points in the DSQIID‐G to identify cognitive changes in DS with high sensitivity and subsequently confirm the clinical suspicion with fluid DSAD biomarkers. Given the inherent potential for bias in caregiver‐reported scores and potential confounding factors on fluid biomarkers such as kidney function, this combined tool, despite its overall good performance in the current study, should not function as the sole basis of an individual DSAD diagnosis. However, with its applicability and cost effectiveness, it can serve as an efficient screening tool in primary care for identifying individuals with relevant cognitive decline and provide guidance on necessary further differential diagnostic steps. In light of upcoming disease‐modifying treatments, this approach can increase care access for this unique population and help overcome barriers such as diagnostic overshadowing.

We want to acknowledge certain limitations. Both cohorts were recruited at the same institution, rendering findings somewhat limited in generalizability toward other demographics and languages, which may contribute to variability in optimal cut‐off scores for DSQIID‐G as well as biomarkers. Future assessments of the DSQIID should ideally aim to validate our findings in larger, multicentric cohorts to capture greater demographic and clinical diversity as well as translation effects. While pooling both cohorts allowed for more stable results in a larger sample, it may also obscure cohort‐specific nuances because the Youden index, although standard, prioritizes overall accuracy without considering context‐specific trade‐offs. Further, *z* scores were obtained from a healthy euploid cohort; future efforts should include the establishment of a healthy DS cohort for reference. Finally, practical measures of implementation are warranted on how to integrate the test and biomarker assessment into clinical workflows, balancing diagnostic yield with cost and feasibility especially for medical providers in primary care, without access to specialized diagnostics and equipment. Additionally, we focused on the widely available fluid biomarkers NfL and GFAP, not including emerging biomarkers such as phosphorylated tau (p‐tau)217. Despite its applicability in sAD, the limited and conflicting data on p‐tau217 in DSAD^28^, ^29^ as well as its unavailability outside the United States made it less suitable for our current investigation of a pragmatic, combined screening tool. Nevertheless, future studies will certainly need to assess its clinical applicability in DSAD.

In conclusion, this study confirms the potential of the DSQIID‐G as a screening tool for detecting emerging cognitive or functional decline in DS adults at risk of developing AD. In an independent cohort, it demonstrated reproducible optimal thresholds while retaining sensitivity to sample characteristics. The addition of blood biomarkers NfL and GFAP consistently enhanced discriminatory power, introducing disease‐specific context of AD pathophysiology, and underscoring the promise of a practical multimodal approach. Such integration into workflows of primary care could facilitate early identification of DS individuals in need of a comprehensive diagnostic workup. This becomes increasingly important with emerging anti‐amyloid therapies, for which early referral could result in meaningful benefit from administration of disease‐modifying interventions.

## CONFLICT OF INTEREST STATEMENT

O.W., C.G., E.W., K.S., C.P., A.S., L.M., A.J., S.H., G.H., and G.N. declare no conflict of interest.

J.L. reports speaker fees from Bayer Vital, Biogen, EISAI, Lilly, TEVA, Bial, Zambon, Esteve, Merck, and Roche; consulting fees from Axon Neuroscience, EISAI, Alnylam, and Biogen; author fees from Thieme medical publishers and W. Kohlhammer GmbH medical publishers; and is inventor in a patent “Oral Phenylbutyrate for Treatment of Human 4‐Repeat Tauopathies” (PCT/EP2024/053388) filed by LMU Munich. In addition, he reports compensation for serving as chief medical officer for MODAG GmbH, is beneficiary of the phantom share program of MODAG GmbH, and is inventor on a patent “Pharmaceutical Composition and Methods of Use” (EP 22 159 408.8) filed by MODAG GmbH, all activities outside the submitted work. H.T. received honoraria for acting as a consultant/speaker and/or for attending events sponsored by Alexion, Bayer, Biogen, Bristol‐Myers Squibb, Celgene, Diamed, Fresenius, Fujirebio, GlaxoSmithKline, Horizon, Janssen‐Cilag, Merck, Novartis, Roche, Sanofi‐Genzyme, Siemens, Teva, and Viatris (all not related to the topic of the study). Author disclosures are available in the supporting information.

## CONSENT STATEMENT

All participants and respective caregivers included in this analysis were recruited through the outpatient clinic of the department of neurology at LMU University Hospital Munich, Germany, providing written informed consent, either themselves or by proxy, prior to study inclusion. The study conduction adhered to the Declaration of Helsinki in its latest revision and was approved by the local institutional review board (#535‐15 and #17‐126).

## Supporting information



Supporting Information

Supporting Information
