# Supplementary material for: Combining blood biomarkers and the German version of the Dementia Screening Questionnaire for Individuals with Intellectual Disabilities (DSQIID‐G) for diagnosing cognitive decline in Down syndrome
Source: Alzheimers Dement. 2026 Mar 20;22(3):e71296. doi: 10.1002/alz.71296 (PMC13093827; doi:10.1002/alz.71296)

SUPPLEMENTAL MATERIALS
for

"Combining blood biomarkers and the German version of the dementia screening questionnaire for individuals with intellectual disabilities (DSQIID-G) for diagnosing cognitive decline in Down Syndrome"

S-Table 1: P-values of the Kruskal-Wallis-Test with Bejamin-Hochberg for multiple testing correction for investigation of cross-sectional DSQIID-G scores per diagnostic group in the validation and whole cohort.

S-Table 2: Youden indices for Receiver Operating Characteristic Analysis for DSQIID-G in the validation cohort detailing sDS+pDS vs aDS

S-Table 3: Youden indices for Receiver Operating Characteristic Analysis for DSQIID-G in the validation cohort detailing sDS+pDS vs the remaining cohort including aDS

S-Table 4: Youden indices for Receiver Operating Characteristic Analysis for DSQIID-G in the whole cohort detailing sDS+pDS vs aDS

S-Table 5: Youden indices for Receiver Operating Characteristic Analysis for DSQIID-G in the whole cohort detailing sDS+pDS vs the remaining cohort including aDS

S-Table 6: Youden indices for Receiver Operating Characteristic Analysis for DSQIID-G in the validation cohort detailing sDS vs CDsecond

S-Table 7: Youden indices for Receiver Operating Characteristic Analysis for DSQIID-G in the whole cohort detailing sDS vs CDsecond

S-Table 8: Youden indices for Receiver Operating Characteristic Analysis for NfL in the validation cohort detailing sDS+pDS vs aDS

S-Table 9: Youden indices for Receiver Operating Characteristic Analysis for NfL in the validation cohort detailing sDS+pDS vs the remaining cohort including aDS

S-Table 10: Youden indices for Receiver Operating Characteristic Analysis for NfL in the whole cohort detailing sDS+pDS vs aDS

S-Table 11: Youden indices for Receiver Operating Characteristic Analysis for NfL in the whole cohort detailing sDS+pDS vs the remaining cohort including aDS

S-Table 12: Youden indices for Receiver Operating Characteristic Analysis for GFAP in the validation cohort detailing sDS+pDS vs aDS

S-Table 13: Youden indices for Receiver Operating Characteristic Analysis for GFAP in the validation cohort detailing sDS+pDS vs the remaining cohort including aDS

S-Table 14: Youden indices for Receiver Operating Characteristic Analysis for GFAP in the whole cohort detailing sDS+pDS vs aDS

S-Table 15: Youden indices for Receiver Operating Characteristic Analysis for GFAP in the whole cohort detailing sDS+pDS vs the remaining cohort including aDS

S-Table 16: Results from logistic regression investigating discriminatory powers via AUC in the validation cohort using logged values for NfL and GFAP

S-Table 17: Results from logistic regression investigating discriminatory powers via AUC in the whole cohort using logged values for NfL and GFAP

S-Table 18: Conditional biomarker cutoffs depending on DSQIID-G score (<7 / ≥7) derived from multivariate logistic regression with confidence intervals in the whole cohort detailing sDS+pDS vs the remaining cohort including aDS for NfL and GFAP

S-Table 19: Results from logistic regression investigating discriminatory powers via AUC in the validation cohort using z-scores for NfL and GFAP

S-Table 20: Results from logistic regression investigating discriminatory powers via AUC in the whole cohort using z-scores for NfL and GFAP

S-Figure 1: ROC analysis via logistic regression for the validation (left) and whole (right) cohort investigating the predictive potential of DSQIID-G, DSQIID-G + zNfL and DSQIID-G + zGFAP for sDS vs aDS (A&B) and sDS vs the rest of the cohort, (C&D) alongside the corresponding AUC estimates.

S-Figure 2: ROC analysis via logistic regression for the validation (left) and whole (right) cohort investigating the predictive potential of DSQIID-G, DSQIID-G + zNfL and DSQIID-G + zGFAP for sDS+pDS vs aDS (A&B) and sDS+pDS vs the rest of the cohort, (C&D) alongside the corresponding AUC estimates.

S-Figure 3: Correlations of relative CAMCOG performance in % with DSQIID-G scores as well as NFL and GFAP levels in the validation (left) and whole (right) cohort according to diagnostic groups.

S-Table 1: P-values of the Kruskal-Wallis-Test with Bejamin-Hochberg correction for multiple testing for the comparison of cross-sectional DSQIID-G scores per diagnostic group in the validation (A) and whole (B) cohort. Abbreviations: aDS, asymptomatic Down Syndrome; CDsecond, secondary cognitive decline not due to dementia, CDunclass, secondary cognitive decline of unclear etiology; pDS, presymptomatic DSAD; sDS, symptomatic DSAD.

A	***aDS***	***pDS***	***sDS***	***CDsecond***
pDS	*0.062*			
sDS	*0.000*	*0.475*		
CDsecond	*0.000*	*0.405*	*0.933*	
CDunclass	*0.011*	*0.786*	*0.806*	*0.786*

B	*aDS*	*pDS*	*sDS*	*CDsecond*
pDS	*0.034*			
sDS	*0.000*	*0.004*		
CDsecond	*0.000*	*0.009*	*0.334*	
CDunclass	*0.000*	*0.009*	*0.336*	*0.970*

S-Table 2: Youden indices for Receiver Operating Characteristic Analysis for DSQIID-G in the validation cohort detailing sDS+pDS vs aDS.

**threshold**	**sensitivity**	**specificity**	**youden_index**
-Inf	1	0	0
0.50	0.94	0.33	0.27
1.50	0.94	0.37	0.31
2.50	0.94	0.43	0.38
3.50	0.89	0.59	0.48
4.50	0.89	0.63	0.52
5.50	0.89	0.67	0.56
6.50	0.89	0.72	0.61
7.50	0.89	0.74	0.63
8.50	0.89	0.76	0.65
10.00	0.89	0.78	0.67
11.50	0.83	0.80	0.64
13.00	0.83	0.85	0.68
14.50	0.83	0.87	0.70
15.50	0.78	0.87	0.65
17.00	0.78	0.89	0.67
18.50	0.61	0.91	0.52
20.00	0.61	0.93	0.55
21.50	0.56	0.98	0.53

23.50	0.50	1.00	0.50
25.50	0.44	1.00	0.44
26.50	0.39	1.00	0.39
28.00	0.33	1.00	0.33
32.00	0.22	1.00	0.22
36.50	0.11	1.00	0.11
40.50	0.06	1.00	0.06
Inf	0	1	0

S-Table 3: Youden indices for Receiver Operating Characteristic Analysis for DSQIID-G in the validation cohort detailing sDS+pDS vs the remaining cohort including aDS.

threshold	sensitivity	specificity	youden_index
-Inf	1	0	0
0.50	0.94	0.25	0.19
1.50	0.94	0.28	0.23
2.50	0.94	0.33	0.28
3.50	0.89	0.45	0.34
4.50	0.89	0.48	0.37
5.50	0.89	0.52	0.41
6.50	0.89	0.55	0.44
7.50	0.89	0.57	0.46
8.50	0.89	0.58	0.47
10.00	0.89	0.62	0.51
11.50	0.83	0.63	0.47
13.00	0.83	0.68	0.52
14.50	0.83	0.70	0.53
15.50	0.78	0.70	0.48
17.00	0.78	0.72	0.49
18.50	0.61	0.73	0.34
20.00	0.61	0.80	0.41
21.50	0.56	0.85	0.41
22.50	0.50	0.88	0.38
24.00	0.50	0.90	0.40
25.50	0.44	0.90	0.34
26.50	0.39	0.90	0.29
28.00	0.33	0.90	0.23
29.50	0.22	0.92	0.14
31.00	0.22	0.93	0.16
32.50	0.22	0.95	0.17

34.00	0.22	0.97	0.19
36.50	0.11	0.98	0.09
38.50	0.06	0.98	0.04
41.00	0.06	1.00	0.06
Inf	0	1	0

S-Table 4: Youden indices for Receiver Operating Characteristic Analysis for DSQIID-G in the whole cohort detailing sDS+pDS vs aDS.

**threshold**	**sensitivity**	**specificity**	**youden_index**
-Inf	1	0	0
0.50	0.95	0.31	0.27
1.50	0.95	0.38	0.33
2.50	0.95	0.47	0.42
3.50	0.90	0.57	0.48
4.50	0.88	0.62	0.50
5.50	0.88	0.67	0.56
6.50	0.86	0.73	0.59
7.50	0.81	0.75	0.56
8.50	0.79	0.78	0.56
10.00	0.79	0.79	0.57
11.50	0.74	0.80	0.54
12.50	0.67	0.82	0.49
13.50	0.67	0.84	0.51
14.50	0.64	0.89	0.53
15.50	0.62	0.89	0.51
16.50	0.62	0.90	0.52
17.50	0.60	0.90	0.49
18.50	0.52	0.92	0.45
20.00	0.50	0.93	0.43
21.50	0.48	0.99	0.46
23.00	0.45	1.00	0.45
24.50	0.43	1.00	0.43
25.50	0.38	1.00	0.38
26.50	0.36	1.00	0.36
28.00	0.33	1.00	0.33
31.00	0.29	1.00	0.29
33.50	0.26	1.00	0.26
34.50	0.21	1.00	0.21
36.00	0.17	1.00	0.17
37.50	0.12	1.00	0.12

40.50	0.07	1.00	0.07
44.50	0.05	1.00	0.05
47.50	0.02	1.00	0.02
Inf	0	1	0

S-Table 5: Youden indices for Receiver Operating Characteristic Analysis for DSQIID-G in the whole cohort detailing sDS+pDS vs the remaining cohort including aDS.

threshold	sensitivity	specificity	youden_index
-Inf	1	0	0
0.50	0.95	0.23	0.18
1.50	0.95	0.28	0.23
2.50	0.95	0.34	0.30
3.50	0.90	0.42	0.32
4.50	0.88	0.45	0.33
5.50	0.88	0.49	0.37
6.50	0.86	0.53	0.39
7.50	0.81	0.57	0.38
8.50	0.79	0.60	0.38
10.00	0.79	0.62	0.41
11.50	0.74	0.64	0.38
12.50	0.67	0.66	0.33
13.50	0.67	0.70	0.36
14.50	0.64	0.74	0.38
15.50	0.62	0.74	0.36
16.50	0.62	0.75	0.36
17.50	0.60	0.75	0.34
18.50	0.52	0.76	0.29
19.50	0.50	0.81	0.31
20.50	0.50	0.82	0.32
21.50	0.48	0.87	0.35
22.50	0.45	0.89	0.35
23.50	0.45	0.91	0.36
24.50	0.43	0.91	0.34
25.50	0.38	0.91	0.29
26.50	0.36	0.91	0.27
27.50	0.33	0.91	0.24
28.50	0.33	0.93	0.26
29.50	0.29	0.94	0.23
31.00	0.29	0.95	0.24
32.50	0.29	0.96	0.24

33.50	0.26	0.97	0.23
34.50	0.21	0.97	0.18
36.00	0.17	0.98	0.15
37.50	0.12	0.98	0.10
38.50	0.07	0.98	0.06
40.00	0.07	0.99	0.06
42.00	0.07	1.00	0.07
44.50	0.05	1.00	0.05
47.50	0.02	1.00	0.02
Inf	0	1	0

S-Table 6: Youden indices for Receiver Operating Characteristic Analysis for DSQIID-G in the validation cohort detailing sDS vs CDsecond

**threshold**	**sensitivity**	**specificity**	**youden**
-Inf	1	0	0
1.50	0.93	0.00	-0.07
7.50	0.87	0.00	-0.13
15.00	0.87	0.10	-0.03
18.50	0.67	0.10	-0.23
20.00	0.67	0.40	0.07
21.50	0.60	0.50	0.10
23.50	0.53	0.60	0.13
25.50	0.47	0.60	0.07
27.50	0.40	0.60	0.00
29.50	0.27	0.70	-0.03
31.50	0.27	0.80	0.07
34.00	0.27	0.90	0.17
36.50	0.13	1.00	0.13
40.50	0.07	1.00	0.07
Inf	0	1	0

S-Table 7: Youden indices for Receiver Operating Characteristic Analysis for DSQIID-G in the whole cohort detailing sDS vs CDsecond

**threshold**	**sensitivity**	**specificity**	**youden**
-Inf	1.00	0.00	0.00
1.50	0.97	0.00	-0.03
3.50	0.94	0.00	-0.06
5.50	0.91	0.00	-0.09

7.50	0.91	0.09	0.00
8.50	0.88	0.18	0.06
10.00	0.88	0.23	0.10
11.50	0.84	0.27	0.12
13.00	0.81	0.32	0.13
15.50	0.78	0.32	0.10
17.50	0.75	0.32	0.07
18.50	0.66	0.32	-0.03
20.00	0.63	0.45	0.08
21.50	0.59	0.50	0.09
22.50	0.56	0.59	0.15
23.50	0.56	0.64	0.20
24.50	0.53	0.64	0.17
25.50	0.47	0.64	0.11
27.00	0.44	0.64	0.07
28.50	0.44	0.68	0.12
29.50	0.38	0.77	0.15
31.50	0.38	0.82	0.19
33.50	0.34	0.86	0.21
34.50	0.28	0.86	0.14
36.00	0.22	0.95	0.17
37.50	0.16	0.95	0.11
39.50	0.09	0.95	0.05
42.00	0.09	1.00	0.09
44.50	0.06	1.00	0.06
47.50	0.03	1.00	0.03
Inf	0.00	1.00	0.00

S-Table 8: Youden indices for Receiver Operating Characteristic Analysis for NfL in the validation cohort detailing sDS+pDS vs aDS

threshold	sensitivity	specificity	youden
-Inf	1	0	0
3.24	1.00	0.02	0.02
3.74	1.00	0.05	0.05
4.51	1.00	0.07	0.07
5.42	1.00	0.10	0.10
5.92	1.00	0.12	0.12
6.03	1.00	0.14	0.14
6.89	1.00	0.17	0.17
7.86	1.00	0.19	0.19
8.86	1.00	0.24	0.24

9.86	1.00	0.26	0.26
10.20	1.00	0.29	0.29
10.70	1.00	0.31	0.31
11.50	1.00	0.33	0.33
12.20	1.00	0.38	0.38
12.50	1.00	0.40	0.40
12.65	1.00	0.43	0.43
12.85	1.00	0.45	0.45
13.20	1.00	0.50	0.50
13.70	0.94	0.50	0.44
14.50	0.94	0.52	0.46
15.30	0.94	0.55	0.49
15.80	0.94	0.57	0.51
16.50	0.88	0.60	0.47
17.35	0.88	0.64	0.52
18.15	0.88	0.67	0.54
19.30	0.88	0.69	0.57
20.35	0.88	0.71	0.59
21.35	0.88	0.74	0.61
22.05	0.88	0.76	0.64
22.55	0.88	0.79	0.66
23.50	0.88	0.81	0.68
24.25	0.88	0.83	0.71
25.40	0.88	0.86	0.73
27.15	0.81	0.88	0.69
28.20	0.75	0.88	0.63
30.10	0.75	0.90	0.65
32.90	0.69	0.90	0.59
37.00	0.69	0.93	0.62
41.20	0.63	0.93	0.55
43.85	0.56	0.93	0.49
45.35	0.56	0.95	0.51
47.65	0.50	0.95	0.45
50.95	0.44	0.95	0.39
53.00	0.38	0.95	0.33
59.00	0.38	0.98	0.35
64.40	0.31	0.98	0.29
67.70	0.25	0.98	0.23
75.45	0.19	0.98	0.16
87.30	0.13	0.98	0.10
97.15	0.06	0.98	0.04
110.00	0.06	1.00	0.06

Inf	0	1	0

S-Table 9: Youden indices for Receiver Operating Characteristic Analysis for NfL in the validation cohort detailing sDS+pDS vs the remaining cohort including aDS

threshold	sensitivity	specificity	youden
-Inf	1	0	0
3.24	1.00	0.04	0.04
3.74	1.00	0.06	0.06
4.51	1.00	0.08	0.08
5.42	1.00	0.10	0.10
5.92	1.00	0.12	0.12
6.03	1.00	0.15	0.15
6.89	1.00	0.17	0.17
7.86	1.00	0.19	0.19
8.86	1.00	0.23	0.23
9.86	1.00	0.25	0.25
10.20	1.00	0.29	0.29
10.70	1.00	0.31	0.31
11.50	1.00	0.33	0.33
12.20	1.00	0.37	0.37
12.50	1.00	0.38	0.38
12.65	1.00	0.40	0.40
12.85	1.00	0.42	0.42
13.20	1.00	0.46	0.46
13.70	0.94	0.46	0.40
14.30	0.94	0.48	0.42
14.80	0.94	0.50	0.44
15.30	0.94	0.52	0.46
15.80	0.94	0.54	0.48
16.50	0.88	0.56	0.43
17.35	0.88	0.60	0.47
18.15	0.88	0.62	0.49
19.30	0.88	0.63	0.51
20.35	0.88	0.65	0.53
21.35	0.88	0.67	0.55
22.05	0.88	0.69	0.57
22.55	0.88	0.71	0.59
23.40	0.88	0.73	0.61
23.90	0.88	0.75	0.63
24.25	0.88	0.77	0.64

25.40	0.88	0.79	0.66
27.15	0.81	0.81	0.62
28.20	0.75	0.81	0.56
29.50	0.75	0.83	0.58
31.20	0.75	0.85	0.60
32.35	0.69	0.85	0.53
33.45	0.69	0.87	0.55
36.00	0.69	0.88	0.57
39.00	0.69	0.90	0.59
41.15	0.63	0.90	0.53
42.35	0.63	0.92	0.55
43.85	0.56	0.92	0.49
45.35	0.56	0.94	0.50
47.65	0.50	0.94	0.44
50.95	0.44	0.94	0.38
53.00	0.38	0.94	0.32
59.00	0.38	0.96	0.34
64.40	0.31	0.96	0.27
67.70	0.25	0.96	0.21
75.45	0.19	0.96	0.15
87.25	0.13	0.96	0.09
94.25	0.13	0.98	0.11
97.15	0.06	0.98	0.04
110.00	0.06	1.00	0.06
Inf	0	1	0

S-Table 10: Youden indices for Receiver Operating Characteristic Analysis for NfL in the whole cohort detailing sDS+pDS vs aDS

threshold	sensitivity	specificity	youden
-Inf	1	0	0
3.24	1.00	0.01	0.01
3.74	1.00	0.03	0.03
4.51	1.00	0.04	0.04
5.42	1.00	0.05	0.05
5.92	1.00	0.07	0.07
6.03	1.00	0.08	0.08
6.18	1.00	0.09	0.09
6.49	1.00	0.12	0.12
6.77	1.00	0.13	0.13
7.20	1.00	0.14	0.14
7.63	1.00	0.16	0.16

7.86	1.00	0.17	0.17
8.18	1.00	0.20	0.20
8.59	1.00	0.21	0.21
8.84	1.00	0.22	0.22
9.09	1.00	0.24	0.24
9.38	1.00	0.25	0.25
9.58	1.00	0.26	0.26
9.79	1.00	0.28	0.28
9.93	1.00	0.29	0.29
10.20	1.00	0.30	0.30
10.50	1.00	0.32	0.32
10.80	1.00	0.33	0.33
11.30	1.00	0.34	0.34
11.80	1.00	0.36	0.36
12.10	1.00	0.38	0.38
12.30	1.00	0.39	0.39
12.45	1.00	0.41	0.41
12.55	0.97	0.42	0.39
12.65	0.97	0.45	0.42
12.85	0.97	0.46	0.43
13.20	0.97	0.49	0.46
13.60	0.94	0.49	0.43
13.90	0.94	0.51	0.45
14.05	0.94	0.53	0.47
14.25	0.94	0.54	0.48
14.70	0.94	0.55	0.49
15.25	0.94	0.58	0.52
15.55	0.94	0.59	0.53
15.65	0.94	0.61	0.55
15.85	0.94	0.62	0.56
16.25	0.91	0.63	0.54
16.75	0.91	0.64	0.56
17.10	0.91	0.67	0.58
17.35	0.91	0.68	0.60
17.60	0.91	0.70	0.61
18.15	0.91	0.71	0.62
18.85	0.91	0.72	0.64
19.55	0.91	0.74	0.65
20.35	0.91	0.75	0.66
21.35	0.91	0.76	0.67
22.05	0.91	0.79	0.70

22.55	0.91	0.80	0.71
23.50	0.91	0.82	0.73
24.25	0.91	0.83	0.74
25.15	0.88	0.84	0.72
26.05	0.85	0.84	0.70
26.70	0.82	0.86	0.68
27.55	0.82	0.87	0.69
28.20	0.79	0.87	0.66
28.55	0.79	0.88	0.68
30.25	0.76	0.88	0.65
32.40	0.74	0.88	0.62
33.25	0.74	0.89	0.63
33.75	0.71	0.89	0.60
35.45	0.71	0.91	0.61
37.90	0.68	0.91	0.58
39.45	0.65	0.91	0.55
40.05	0.62	0.91	0.53
41.25	0.59	0.91	0.50
42.45	0.56	0.91	0.47
43.15	0.56	0.92	0.48
44.55	0.53	0.92	0.45
45.35	0.53	0.93	0.46
45.70	0.50	0.93	0.43
47.95	0.47	0.93	0.40
50.95	0.44	0.93	0.38
53.00	0.41	0.93	0.35
54.20	0.41	0.96	0.37
55.05	0.38	0.96	0.34
59.85	0.35	0.96	0.31
64.40	0.32	0.96	0.28
65.15	0.29	0.97	0.27
67.30	0.26	0.97	0.24
69.85	0.24	0.97	0.21
71.00	0.21	0.97	0.18
73.40	0.18	0.97	0.15
77.85	0.15	0.97	0.12
85.20	0.12	0.97	0.09
91.10	0.09	0.97	0.06
93.20	0.06	0.97	0.03
97.15	0.03	0.97	0.00
110.00	0.03	0.99	0.02
181.00	0.00	0.99	-0.01

Inf	0	1	0

S-Table 11: Youden indices for Receiver Operating Characteristic Analysis for NfL in the whole cohort detailing sDS+pDS vs the remaining cohort including aDS

threshold	sensitivity	specificity	youden
-Inf	1	0	0
3.24	1.00	0.02	0.02
3.74	1.00	0.03	0.03
4.32	1.00	0.04	0.04
4.81	1.00	0.05	0.05
5.42	1.00	0.06	0.06
5.92	1.00	0.07	0.07
6.03	1.00	0.09	0.09
6.18	1.00	0.10	0.10
6.49	1.00	0.12	0.12
6.77	1.00	0.13	0.13
7.20	1.00	0.14	0.14
7.63	1.00	0.15	0.15
7.86	1.00	0.16	0.16
8.17	1.00	0.18	0.18
8.35	1.00	0.19	0.19
8.59	1.00	0.20	0.20
8.84	1.00	0.21	0.21
9.09	1.00	0.22	0.22
9.38	1.00	0.23	0.23
9.58	1.00	0.24	0.24
9.79	1.00	0.25	0.25
9.93	1.00	0.26	0.26
10.20	1.00	0.28	0.28
10.50	1.00	0.29	0.29
10.80	1.00	0.30	0.30
11.30	1.00	0.31	0.31
11.80	1.00	0.32	0.32
12.10	1.00	0.34	0.34
12.30	1.00	0.35	0.35
12.45	1.00	0.37	0.37
12.55	0.97	0.38	0.35
12.65	0.97	0.40	0.37
12.75	0.97	0.41	0.38
12.90	0.97	0.42	0.39

13.20	0.97	0.44	0.42
13.60	0.94	0.44	0.39
13.85	0.94	0.46	0.41
13.95	0.94	0.47	0.42
14.05	0.94	0.48	0.43
14.25	0.94	0.49	0.44
14.50	0.94	0.51	0.45
14.80	0.94	0.52	0.46
15.25	0.94	0.54	0.48
15.55	0.94	0.55	0.49
15.65	0.94	0.56	0.50
15.85	0.94	0.57	0.51
16.25	0.91	0.58	0.49
16.60	0.91	0.59	0.50
16.85	0.91	0.60	0.51
17.10	0.91	0.62	0.53
17.25	0.91	0.63	0.54
17.40	0.91	0.64	0.55
17.60	0.91	0.65	0.56
18.15	0.91	0.67	0.58
18.80	0.91	0.68	0.59
19.05	0.91	0.69	0.60
19.15	0.91	0.70	0.61
19.60	0.91	0.71	0.62
20.35	0.91	0.72	0.63
21.05	0.91	0.73	0.64
21.70	0.91	0.74	0.65
22.05	0.91	0.76	0.67
22.55	0.91	0.77	0.68
23.40	0.91	0.78	0.69
23.90	0.91	0.79	0.70
24.25	0.91	0.80	0.71
25.15	0.88	0.81	0.69
26.05	0.85	0.81	0.66
26.35	0.82	0.82	0.64
26.75	0.82	0.83	0.65
27.55	0.82	0.84	0.66
28.20	0.79	0.84	0.63
28.55	0.79	0.85	0.64
29.65	0.76	0.85	0.61
31.20	0.76	0.86	0.62
32.35	0.74	0.86	0.59

32.95	0.74	0.87	0.60
33.25	0.74	0.88	0.61
33.75	0.71	0.88	0.58
35.45	0.71	0.89	0.59
37.45	0.68	0.89	0.57
38.45	0.68	0.90	0.58
39.45	0.65	0.90	0.55
40.05	0.62	0.90	0.52
41.20	0.59	0.90	0.49
42.35	0.59	0.92	0.51
42.45	0.56	0.92	0.48
43.15	0.56	0.93	0.49
44.55	0.53	0.93	0.46
45.35	0.53	0.94	0.47
45.70	0.50	0.94	0.44
47.95	0.47	0.94	0.41
50.95	0.44	0.94	0.38
53.00	0.41	0.94	0.35
54.20	0.41	0.96	0.37
55.05	0.38	0.96	0.34
59.85	0.35	0.96	0.31
64.40	0.32	0.96	0.28
65.15	0.29	0.97	0.26
67.30	0.26	0.97	0.23
69.85	0.24	0.97	0.20
71.00	0.21	0.97	0.18
73.40	0.18	0.97	0.15
77.85	0.15	0.97	0.12
85.20	0.12	0.97	0.09
91.10	0.09	0.97	0.06
93.15	0.06	0.97	0.03
94.25	0.06	0.98	0.04
97.15	0.03	0.98	0.01
110.00	0.03	0.99	0.02
181.00	0.00	0.99	-0.01
Inf	0	1	0

S-Table 12: Youden indices for Receiver Operating Characteristic Analysis for GFAP in the validation cohort detailing sDS+pDS vs aDS

threshold	sensitivity	specificity	youden
-Inf	1	0	0

2.01	1.00	0.02	0.02
2.39	1.00	0.05	0.05
2.60	1.00	0.07	0.07
2.90	1.00	0.10	0.10
3.15	1.00	0.12	0.12
3.33	1.00	0.14	0.14
3.46	1.00	0.17	0.17
3.60	1.00	0.19	0.19
3.89	1.00	0.21	0.21
4.15	1.00	0.24	0.24
4.30	1.00	0.26	0.26
4.52	1.00	0.29	0.29
4.70	1.00	0.31	0.31
4.76	1.00	0.33	0.33
4.85	1.00	0.36	0.36
5.01	1.00	0.38	0.38
5.14	0.93	0.38	0.31
5.21	0.93	0.40	0.33
5.47	0.93	0.43	0.36
5.69	0.93	0.45	0.38
5.88	0.93	0.48	0.40
6.16	0.93	0.50	0.43
6.49	0.93	0.52	0.45
6.73	0.93	0.55	0.48
6.76	0.93	0.57	0.50
6.89	0.93	0.62	0.55
7.04	0.93	0.64	0.57
7.07	0.93	0.67	0.60
7.10	0.93	0.69	0.62
7.34	0.93	0.71	0.64
7.63	0.93	0.74	0.67
7.72	0.93	0.76	0.69
7.92	0.93	0.81	0.74
8.19	0.93	0.83	0.76
8.87	0.93	0.86	0.79
9.57	0.93	0.88	0.81
9.84	0.86	0.88	0.74
10.15	0.86	0.90	0.76
11.10	0.79	0.90	0.69
11.95	0.79	0.93	0.71
12.20	0.71	0.93	0.64

13.35	0.64	0.93	0.57
14.55	0.57	0.93	0.50
15.40	0.57	0.95	0.52
16.05	0.50	0.95	0.45
16.20	0.50	0.98	0.48
16.55	0.43	0.98	0.40
16.95	0.36	0.98	0.33
17.35	0.29	0.98	0.26
18.20	0.21	0.98	0.19
19.15	0.21	1.00	0.21
19.95	0.14	1.00	0.14
24.05	0.07	1.00	0.07
Inf	0	1	0

S-Table 13: Youden indices for Receiver Operating Characteristic Analysis for GFAP in the validation cohort detailing sDS+pDS vs the remaining cohort including aDS

threshold	sensitivity	specificity	youden
-Inf	1	0	0
2.01	1.00	0.02	0.02
2.39	1.00	0.04	0.04
2.56	1.00	0.06	0.06
2.68	1.00	0.08	0.08
2.82	1.00	0.10	0.10
3.00	1.00	0.12	0.12
3.15	1.00	0.13	0.13
3.33	1.00	0.15	0.15
3.45	1.00	0.17	0.17
3.47	1.00	0.19	0.19
3.60	1.00	0.21	0.21
3.89	1.00	0.23	0.23
4.15	1.00	0.25	0.25
4.30	1.00	0.27	0.27
4.45	1.00	0.29	0.29
4.62	1.00	0.31	0.31
4.70	1.00	0.33	0.33
4.75	1.00	0.35	0.35
4.80	1.00	0.37	0.37
4.85	1.00	0.38	0.38
5.01	1.00	0.40	0.40
5.14	0.93	0.40	0.33
5.21	0.93	0.42	0.35

5.47	0.93	0.44	0.37
5.69	0.93	0.46	0.39
5.72	0.93	0.48	0.41
5.89	0.93	0.50	0.43
6.16	0.93	0.52	0.45
6.49	0.93	0.54	0.47
6.73	0.93	0.56	0.49
6.76	0.93	0.58	0.51
6.89	0.93	0.62	0.54
7.04	0.93	0.63	0.56
7.07	0.93	0.65	0.58
7.10	0.93	0.67	0.60
7.34	0.93	0.69	0.62
7.63	0.93	0.71	0.64
7.72	0.93	0.73	0.66
7.92	0.93	0.77	0.70
8.19	0.93	0.79	0.72
8.87	0.93	0.81	0.74
9.57	0.93	0.83	0.76
9.84	0.86	0.83	0.68
10.15	0.86	0.85	0.70
11.10	0.79	0.85	0.63
11.95	0.79	0.87	0.65
12.20	0.71	0.87	0.58
12.80	0.64	0.87	0.51
13.75	0.64	0.88	0.53
14.55	0.57	0.88	0.46
14.85	0.57	0.90	0.48
15.45	0.57	0.92	0.49
16.05	0.50	0.92	0.42
16.20	0.50	0.94	0.44
16.45	0.43	0.94	0.37
16.70	0.43	0.96	0.39
16.95	0.36	0.96	0.32
17.35	0.29	0.96	0.25
18.20	0.21	0.96	0.18
19.15	0.21	0.98	0.20
19.95	0.14	0.98	0.12
24.05	0.07	0.98	0.05
29.55	0.00	0.98	-0.02
Inf	0	1	0

S-Table 14: Youden indices for Receiver Operating Characteristic Analysis for GFAP in the whole cohort detailing sDS+pDS vs aDS

threshold	sensitivity	specificity	youden
-Inf	1	0	0
2.01	1.00	0.01	0.01
2.39	1.00	0.03	0.03
2.60	1.00	0.04	0.04
2.90	1.00	0.05	0.05
3.15	1.00	0.07	0.07
3.33	1.00	0.08	0.08
3.46	1.00	0.09	0.09
3.60	1.00	0.11	0.11
3.86	1.00	0.12	0.12
4.03	1.00	0.13	0.13
4.14	1.00	0.14	0.14
4.23	1.00	0.16	0.16
4.29	1.00	0.17	0.17
4.35	1.00	0.18	0.18
4.44	1.00	0.20	0.20
4.55	1.00	0.21	0.21
4.63	1.00	0.22	0.22
4.69	1.00	0.24	0.24
4.70	1.00	0.25	0.25
4.73	1.00	0.26	0.26
4.77	1.00	0.28	0.28
4.85	1.00	0.30	0.30
4.94	1.00	0.32	0.32
5.01	1.00	0.33	0.33
5.08	1.00	0.34	0.34
5.14	0.97	0.34	0.31
5.18	0.97	0.36	0.32
5.23	0.97	0.37	0.34
5.26	0.97	0.38	0.35
5.28	0.97	0.39	0.36
5.40	0.97	0.41	0.38
5.51	0.97	0.42	0.39
5.55	0.97	0.43	0.40
5.63	0.94	0.43	0.37
5.68	0.94	0.45	0.38
5.70	0.94	0.46	0.40

5.75	0.94	0.47	0.41
5.82	0.91	0.47	0.38
5.95	0.91	0.49	0.39
6.06	0.91	0.50	0.41
6.08	0.91	0.51	0.42
6.10	0.91	0.53	0.43
6.19	0.91	0.54	0.45
6.32	0.91	0.55	0.46
6.51	0.91	0.57	0.47
6.67	0.91	0.58	0.49
6.69	0.91	0.59	0.50
6.73	0.91	0.61	0.51
6.76	0.91	0.62	0.52
6.89	0.91	0.64	0.55
7.04	0.91	0.66	0.56
7.07	0.91	0.67	0.58
7.09	0.91	0.68	0.59
7.12	0.91	0.70	0.60
7.34	0.91	0.71	0.62
7.56	0.91	0.72	0.63
7.64	0.88	0.72	0.60
7.72	0.88	0.74	0.61
7.89	0.88	0.76	0.64
8.05	0.88	0.78	0.65
8.07	0.88	0.79	0.66
8.10	0.88	0.82	0.69
8.19	0.88	0.83	0.70
8.61	0.88	0.84	0.72
9.04	0.84	0.84	0.69
9.30	0.81	0.84	0.65
9.57	0.81	0.86	0.67
9.84	0.78	0.86	0.64
10.15	0.78	0.87	0.65
10.50	0.75	0.88	0.63
10.75	0.72	0.89	0.61
11.35	0.69	0.89	0.58
11.95	0.69	0.91	0.60
12.20	0.66	0.91	0.56
13.35	0.63	0.91	0.53
14.55	0.56	0.91	0.47
15.00	0.56	0.92	0.48
15.60	0.56	0.93	0.50

16.05	0.53	0.93	0.47
16.20	0.50	0.95	0.45
16.50	0.47	0.95	0.42
16.75	0.41	0.95	0.35
16.95	0.38	0.95	0.32
17.35	0.34	0.96	0.30
17.65	0.31	0.96	0.27
17.95	0.28	0.96	0.24
18.50	0.25	0.96	0.21
19.05	0.25	0.97	0.22
19.40	0.22	0.97	0.19
19.95	0.19	0.97	0.16
21.40	0.16	0.97	0.13
22.45	0.13	0.97	0.10
22.70	0.09	0.97	0.07
22.95	0.09	0.99	0.08
23.95	0.06	0.99	0.05
26.30	0.03	0.99	0.02
37.40	0.00	0.99	-0.01
Inf	0	1	0

S-Table 15: Youden indices for Receiver Operating Characteristic Analysis for GFAP in the whole cohort detailing sDS+pDS vs the remaining cohort including aDS

threshold	sensitivity	specificity	youden
-Inf	1	0	0
2.01	1.00	0.01	0.01
2.39	1.00	0.02	0.02
2.56	1.00	0.03	0.03
2.68	1.00	0.04	0.04
2.82	1.00	0.05	0.05
3.00	1.00	0.06	0.06
3.15	1.00	0.07	0.07
3.33	1.00	0.08	0.08
3.45	1.00	0.09	0.09
3.47	1.00	0.10	0.10
3.60	1.00	0.11	0.11
3.78	1.00	0.12	0.12
3.91	1.00	0.13	0.13
4.03	1.00	0.14	0.14
4.14	1.00	0.15	0.15
4.23	1.00	0.16	0.16

4.29	1.00	0.17	0.17
4.35	1.00	0.18	0.18
4.44	1.00	0.19	0.19
4.53	1.00	0.20	0.20
4.56	1.00	0.21	0.21
4.63	1.00	0.22	0.22
4.69	1.00	0.23	0.23
4.70	1.00	0.24	0.24
4.72	1.00	0.25	0.25
4.73	1.00	0.26	0.26
4.77	1.00	0.27	0.27
4.80	1.00	0.28	0.28
4.85	1.00	0.30	0.30
4.94	1.00	0.31	0.31
5.01	1.00	0.32	0.32
5.08	1.00	0.33	0.33
5.14	0.97	0.33	0.30
5.18	0.97	0.34	0.31
5.23	0.97	0.35	0.32
5.26	0.97	0.36	0.33
5.28	0.97	0.37	0.34
5.35	0.97	0.38	0.35
5.45	0.97	0.39	0.36
5.51	0.97	0.40	0.37
5.55	0.97	0.41	0.38
5.59	0.97	0.42	0.39
5.63	0.94	0.42	0.36
5.68	0.94	0.43	0.37
5.70	0.94	0.44	0.38
5.72	0.94	0.45	0.39
5.76	0.94	0.46	0.40
5.82	0.91	0.46	0.37
5.95	0.91	0.47	0.38
6.06	0.91	0.48	0.39
6.08	0.91	0.49	0.40
6.10	0.91	0.51	0.41
6.17	0.91	0.52	0.42
6.26	0.91	0.53	0.43
6.32	0.91	0.54	0.44
6.51	0.91	0.55	0.45
6.67	0.91	0.56	0.46
6.69	0.91	0.57	0.47

6.73	0.91	0.58	0.48
6.76	0.91	0.59	0.49
6.87	0.91	0.61	0.51
7.00	0.91	0.62	0.52
7.04	0.91	0.63	0.53
7.07	0.91	0.64	0.54
7.09	0.91	0.65	0.55
7.12	0.91	0.66	0.56
7.28	0.91	0.67	0.57
7.50	0.91	0.68	0.58
7.56	0.91	0.69	0.59
7.64	0.88	0.69	0.56
7.72	0.88	0.70	0.57
7.89	0.88	0.72	0.59
8.05	0.88	0.73	0.60
8.07	0.88	0.74	0.61
8.10	0.88	0.76	0.63
8.19	0.88	0.77	0.64
8.41	0.88	0.79	0.66
8.59	0.88	0.80	0.67
8.65	0.88	0.81	0.68
8.82	0.88	0.82	0.69
9.04	0.84	0.82	0.66
9.30	0.81	0.82	0.63
9.57	0.81	0.83	0.64
9.82	0.78	0.83	0.61
9.99	0.78	0.84	0.62
10.15	0.78	0.85	0.63
10.50	0.75	0.86	0.61
10.75	0.72	0.87	0.59
11.30	0.69	0.87	0.56
11.85	0.69	0.88	0.57
11.95	0.69	0.89	0.58
12.20	0.66	0.89	0.55
12.80	0.63	0.89	0.51
13.75	0.63	0.90	0.52
14.55	0.56	0.90	0.46
14.85	0.56	0.91	0.47
15.05	0.56	0.92	0.48
15.60	0.56	0.93	0.49
16.05	0.53	0.93	0.46
16.20	0.50	0.94	0.44

16.45	0.47	0.94	0.41
16.65	0.47	0.95	0.42
16.75	0.41	0.95	0.36
16.95	0.38	0.95	0.32
17.35	0.34	0.96	0.30
17.65	0.31	0.96	0.27
17.95	0.28	0.96	0.24
18.50	0.25	0.96	0.21
19.05	0.25	0.97	0.22
19.40	0.22	0.97	0.19
19.95	0.19	0.97	0.16
21.40	0.16	0.97	0.13
22.45	0.13	0.97	0.09
22.70	0.09	0.97	0.06
22.95	0.09	0.98	0.07
23.95	0.06	0.98	0.04
26.30	0.03	0.98	0.01
29.55	0.00	0.98	-0.02
39.25	0.00	0.99	-0.01
Inf	0	1	0

S-Table 16: Results from logistic regression investigating discriminatory powers via AUC in the validation cohort. NfL and GFAP were logged for all analyses. AUC, Area under the curve; LR_Pos, positive Likelihood-Ratio; LR_Neg, negative Likelihood-Ratio.

	Validation cohort				
	AUC	Sensitivity	Specificity	LR_Pos	LR_Neg
**sDS vs aDS**					
DSQIID-G	0.861	0.833	0.881	7.000	0.189
DSQIID-G+NfL	0.944	0.750	1.000	Inf	0.250
DSQIID-G+GFAP	0.956	0.917	0.881	7.700	0.095
**sDS vs Rest of cohort**					
DSQIID-G	0.799	0.833	0.741	3.214	0.225
DSQIID-G+NfL	0.907	0.750	0.963	20.250	0.260
DSQIID-G+GFAP	0.915	1.000	0.704	3.375	0.000
**sDS+pDS vs aDS**					
DSQIID-G	0.871	0.857	0.857	6.000	0.167
DSQIID-G+NfL	0.946	0.857	0.905	9.000	0.158
DSQIID-G+GFAP	0.956	0.929	0.881	7.800	0.081
**sDS+pDS vs Rest of cohort**					
DSQIID-G	0.808	0.857	0.731	3.184	0.195
DSQIID-G+NfL	0.911	1.000	0.692	3.250	0.000

DSQIID-G+GFAP	0.919	1.000	0.731	3.714	0.000

S-Table 17: Results from logistic regression investigating discriminatory powers via AUC in the whole cohort. NfL and GFAP were logged for all analyses. AUC, Area under the curve; LR_Pos, positive Likelihood-Ratio; LR_Neg, negative Likelihood-Ratio.

	**Whole cohort**				
	AUC	Sensitivity	Specificity	LR_Pos	LR_Neg
**sDS vs aDS**					
DSQIID-G	0.888	0.760	0.895	7.220	0.268
DSQIID-G+NfL	0.962	0.920	0.908	9.989	0.088
DSQIID-G+GFAP	0.959	0.880	0.908	9.554	0.132
**sDS vs Rest of cohort**					
DSQIID-G	0.819	0.760	0.774	3.357	0.310
DSQIID-G+NfL	0.932	0.800	0.934	12.114	0.214
DSQIID-G+GFAP	0.922	0.800	0.887	7.067	0.226
**sDS+pDS vs aDS**					
DSQIID-G	0.842	0.781	0.776	3.493	0.282
DSQIID-G+NfL	0.924	0.844	0.908	9.161	0.172
DSQIID-G+GFAP	0.912	0.750	0.961	19.000	0.260
**sDS+pDS vs Rest of cohort**					
DSQIID-G	0.766	0.500	0.929	7.071	0.538
DSQIID-G+NfL	0.899	0.813	0.859	5.746	0.218
DSQIID-G+GFAP	0.887	0.750	0.919	9.281	0.272

S-Table 18: Conditional biomarker cutoffs depending on DSQIID-G score (<7 / ≥ 7) derived from multivariate logistic regression with confidence intervals in the whole cohort detailing sDS+pDS vs the remaining cohort including aDS for NfL and GFAP. CI; confidence interval.

	**DSQIID-G**	**Cut-off (pg/mL)**	**CI_lower**	**CI_upper**	**sensitivity**	**specificity**
**sDS+pDs vs Rest NfL**	< 7	47.07	27.15	125.97	0.81	0.85
	≥ 7	25.40	19.84	33.41	0.81	0.85
**sDS+pDs vs Rest GFAP**	< 7	14.30	9.43	30.31	0.81	0.82
	≥ 7	8.11	6.40	10.36	0.81	0.82

S-Table 19: Results from logistic regression investigating discriminatory powers via AUC in the validation cohort using z-scores for NfL and GFAP. AUC, Area under the curve; LR_Pos, positive Likelihood-Ratio; LR_Neg, negative Likelihood-Ratio.

	Validation cohort				
	AUC	Sensitivity	Specificity	LR_Pos	LR_Neg
**sDS vs aDS**					
DSQIID-G	0.861	0.833	0.881	7.000	0.189
DSQIID-G+zNfL	0.944	0.750	1.000	Inf	0.250
DSQIID-G+zGFAP	0.956	0.917	0.881	7.700	0.095
**sDS vs Rest of cohort**					
DSQIID-G	0.799	0.833	0.741	3.214	0.225
DSQIID-G+zNfL	0.907	0.750	0.963	20.250	0.260
DSQIID-G+zGFAP	0.915	1.000	0.704	3.375	0.000
**sDS+pDS vs aDS**					
DSQIID-G	0.871	0.857	0.857	6.000	0.167
DSQIID-G+zNfL	0.946	0.857	0.905	9.000	0.158
DSQIID-G+zGFAP	0.956	0.929	0.881	7.800	0.081
**sDS+pDS vs Rest of cohort**					
DSQIID-G	0.808	0.857	0.731	3.184	0.195
DSQIID-G+zNfL	0.911	1.000	0.692	3.250	0.000
DSQIID-G+zGFAP	0.919	1.000	0.731	3.714	0.000

S-Table 20: Results from logistic regression investigating discriminatory powers via AUC in the whole cohort using z-scores for NfL and GFAP. AUC, Area under the curve; LR_Pos, positive Likelihood-Ratio; LR_Neg, negative Likelihood-Ratio.

	Whole cohort				
	AUC	Sensitivity	Specificity	LR_Pos	LR_Neg
**sDS vs aDS**					
DSQIID-G	0.888	0.760	0.895	7.220	0.268
DSQIID-G+zNfL	0.998	1.000	0.987	76.000	0.000
DSQIID-G+zGFAP	1.000	1.000	1.000	Inf	0.000
**sDS vs Rest of cohort**					
DSQIID-G	0.821	0.760	0.781	3.470	0.307
DSQIID-G+zNfL	0.982	0.960	0.943	16.800	0.042
DSQIID-G+zGFAP	0.994	1.000	0.952	21.000	0.000
**sDS+pDS vs aDS**					
DSQIID-G	0.842	0.781	0.776	3.493	0.282
DSQIID-G+zNfL	0.924	0.844	0.908	9.161	0.172

DSQIID-G+zGFAP	0.912	0.750	0.961	19.000	0.260
**sDS+pDS vs Rest of cohort**					
DSQIID-G	0.766	0.500	0.929	7.071	0.538
DSQIID-G+zNfL	0.899	0.813	0.859	5.746	0.218
DSQIID-G+zGFAP	0.887	0.750	0.919	9.281	0.272

S-Figure 1: ROC analysis via logistic regression for the validation (left) and whole (right) cohort investigating the predictive potential of DSQIID-G, DSQIID-G + zNfL and DSQIID-G + zGFAP for sDS vs aDS (A&B) and sDS vs the rest of the cohort, (C&D) alongside the corresponding AUC estimates.

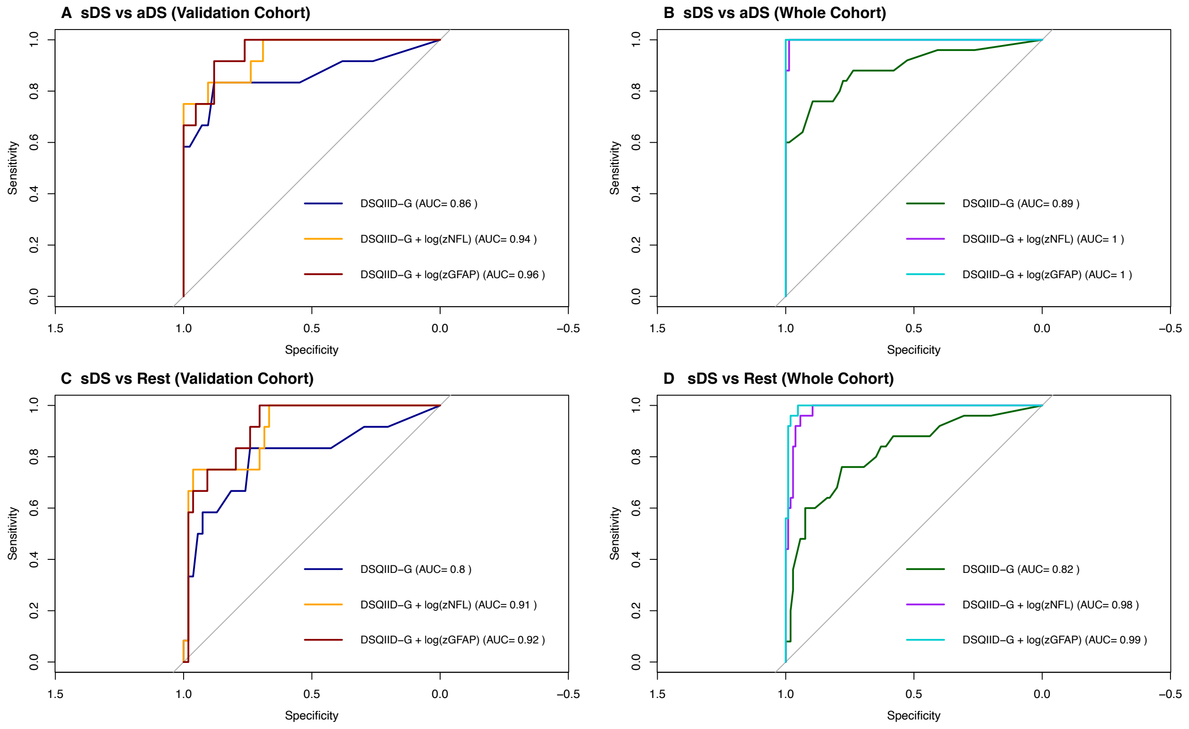

S-Figure 2: ROC analysis via logistic regression for the validation (left) and whole (right) cohort investigating the predictive potential of DSQIID-G, DSQIID-G + zNfL and DSQIID-G + zGFAP for sDS+pDS vs aDS (A&B) and sDS+pDS vs the rest of the cohort, (C&D) alongside the corresponding AUC estimates.

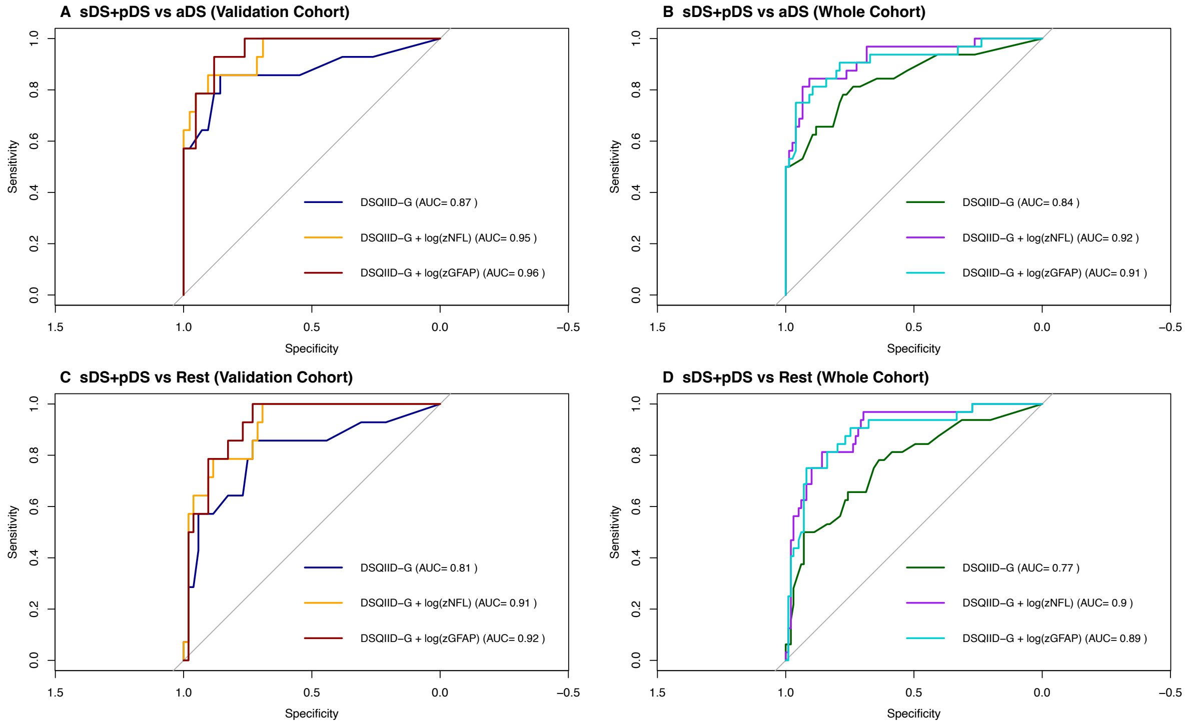

S-Figure 3: Correlations of relative CAMCOG performance in % with DSQIID-G scores as well as NfL and GFAP levels in the validation (left) and whole (right) cohort according to diagnostic groups.

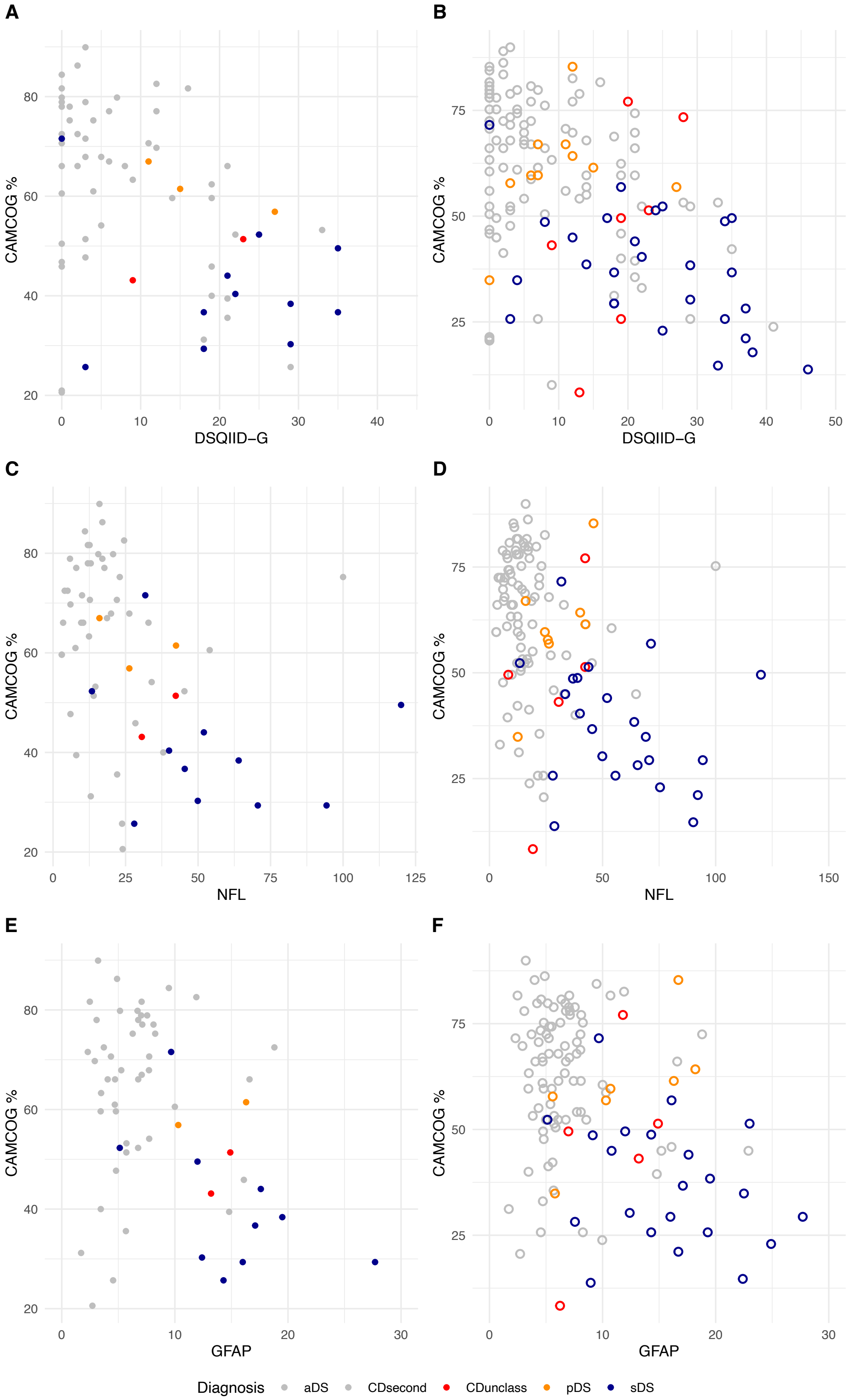

Supplement: Supplementary file 1 — Supporting Information [file ALZ-22-e71296-s002.pdf]
